# Recent advances in bioengineering and functional applications of microbial biocomposites: integrating bacterial cellulose, fungal mycelium and synthetic biology

**DOI:** 10.1007/s11274-026-05021-w

**Published:** 2026-05-19

**Authors:** Marli Camassola, Rosmary Nichele Brandalise

**Affiliations:** 1 Laboratory of Enzymes and Biomasses, Institute of Biotechnology, Caxias do Sul, University of Caxias do Sul, São Paulo, 95070-560 Brazil; 2 Laboratory of Materials, Caxias do Sul, University of Caxias do Sul, São Paulo, 95070-560 Brazil

**Keywords:** Microbial biocomposites, Bacterial cellulose, Fungal mycelium, Synthetic biology, 3D bioprinting, Sustainable biomaterials

## Abstract

The development of microbial biocomposites represents a promising frontier in the design of sustainable, multifunctional, and environmentally friendly materials. This minireview synthesises recent advances (2020–2026) in the production, modification, and application of biocomposites derived from microorganisms, with a focus on bacterial cellulose (BC) and fungal mycelium. It explores their synergies with emerging approaches in synthetic biology, 3D printing, and mineral functionalisation. Key microbial systems such as *Komagataeibacter* spp. and *Ganoderma* spp. are discussed, alongside structural and functional engineering strategies including in situ hydroxyapatite mineralisation, incorporation of plant fibres, and the addition of functional nanomaterials such as graphene oxide. The review further highlights the integration of these materials into high-value applications, including osteogenic scaffolds, self-healing living materials, biodegradable packaging, and environmental remediation systems. Finally, it addresses current regulatory and technical challenges related to industrial scalability, functional stability, and batch-to-batch standardisation. This article aims to provide a critical and comprehensive perspective for researchers and professionals in applied microbiology, materials science, and industrial biotechnology, emphasising the potential of microbial biocomposites as a convergent platform at the interface of sustainability, functional innovation, and bioinspired design.

**Graphical Abstract:**

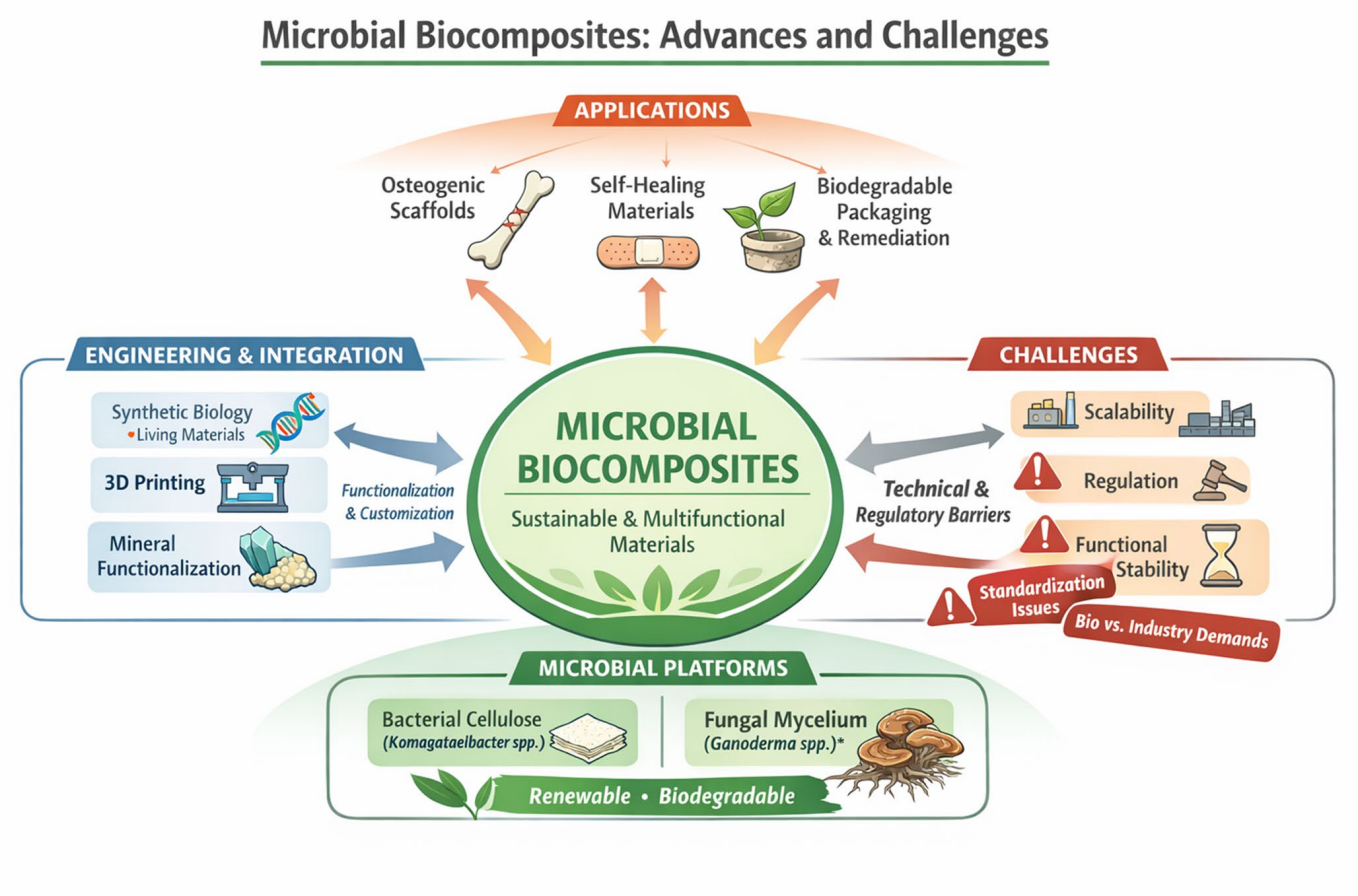

## Introduction

Microbial biocomposites are materials formed by the combination of biologically produced components - such as bacterial cellulose or fungal mycelium - with natural or engineered matrices, resulting in structures whose properties arise from the interaction between these biological elements and other reinforcing phases. Unlike conventional composites derived from petrochemical sources, microbial biocomposites are generated through biological growth processes, which can confer characteristics such as renewability, biodegradability, and the potential for functional responsiveness to environmental stimuli.

The growing demand for sustainable and circular materials has driven an increasing number of studies focused on biocomposites produced by microorganisms, motivated not only by the renewability and biodegradability of these materials, but also by the need to reduce dependence on fossil-based synthetic polymers and to mitigate the severe environmental impacts associated with persistent plastic waste in the biosphere. Recent studies identify natural polymers, such as bacterial cellulose and fungal mycelium-based materials, as emerging platforms for high-performance materials with low ecological impact. (Castro-Dominguez et al. 2025; Subramanian et al. 2025).

Bacterial cellulose (BC), produced primarily by bacteria of the genus *Komagataeibacter*, has attracted considerable attention due to its high purity, elevated crystallinity, and remarkable mechanical strength, as well as its highly functionalizable surface, which enables chemical or physical modifications tailored to specific applications. These properties make BC a promising candidate for applications ranging from bioplastics and sustainable packaging to functional films and active surfaces capable of incorporating functionalities such as sensing or antimicrobial activity, as demonstrated in recently developed biological materials that retain living characteristics and exhibit reversible responses to environmental stimuli (Agha and Katı 2025).

In turn, mycelium-based materials, structured by three-dimensional networks of hyphae bound to lignocellulosic substrates, offer distinctive properties, including low density, thermal and acoustic insulation, biodegradability, and the capacity to grow into moulded forms using residual biomass. Recent reviews highlight the potential of these mycelium-based composites (MBCs) as sustainable alternatives in sectors such as construction, packaging, and industrial design, while also emphasising significant technical challenges, including mechanical inconsistencies, production scalability, and the need for standardised manufacturing protocols (Singh et al. 2025).

In recent years, the biological synthesis of these materials has converged with approaches in synthetic biology and metabolic engineering, paving the way for the creation of living materials (ELMs) — living or semi-living materials capable of responding to environmental stimuli, self-repair, and interacting with biological or abiotic systems. This frontier extends beyond traditional reviews by focusing not only on the static properties of biocomposites, but also on their dynamic capacity for functional adaptation over time. Such an advancement goes beyond simple biodegradable materials and requires new frameworks for biotechnological control and performance criteria that are not yet fully established in the literature (Wattanavichean et al. 2025).

In summary, although recent literature points to significant advances in the production and characterisation of microbial biocomposites — such as mycelium biocomposites reinforced with bacterial cellulose, which exhibit tunable mechanical properties and potential for structural or insulating panels in construction and other sectors (*Trametes versicolor* reinforced with BC) (Elsacker et al. 2021) — as well as the versatility of bacterial cellulose as a functional membrane in food packaging and other selective barrier applications (Çalhan and Hasanoğlu 2026), a substantial gap still remains in integrated analyses that articulate biological foundations, materials engineering, functional performance, and the challenges of scalability and functional stability under real-use conditions.

Mycelium-based materials, for example, have been highlighted as promising for sustainable thermal insulation in buildings due to their low thermal conductivity, comparable to polymeric foams, and low embodied energy (Motamedi et al. 2025), while BC composites have been explored in active packaging membranes capable of modulating gas and vapour transport. However, most existing reviews focus on isolated properties, such as biodegradability or mechanical strength, without critically addressing the challenges of industrial reproducibility, process standardisation, and the integration of adaptive functionalities, such as environmental responsiveness or self-repair, which are essential for the adoption of these materials in advanced technological applications (Pertile et al. 2025).

In this context, the present work distinguishes itself by proposing an integrated and critical analysis of these microbial biocomposites, articulating both their applied potential in packaging, construction, and functional devices and the technological and biotechnological challenges that must be overcome. The aim is to provide a robust foundation for the development of multifunctional, sustainable, and technically viable materials at industrial scale.

## Bacterial cellulose: microbial synthesis and structural characteristics

### Microbial producers and biosynthetic pathways

**Fig. 1:**
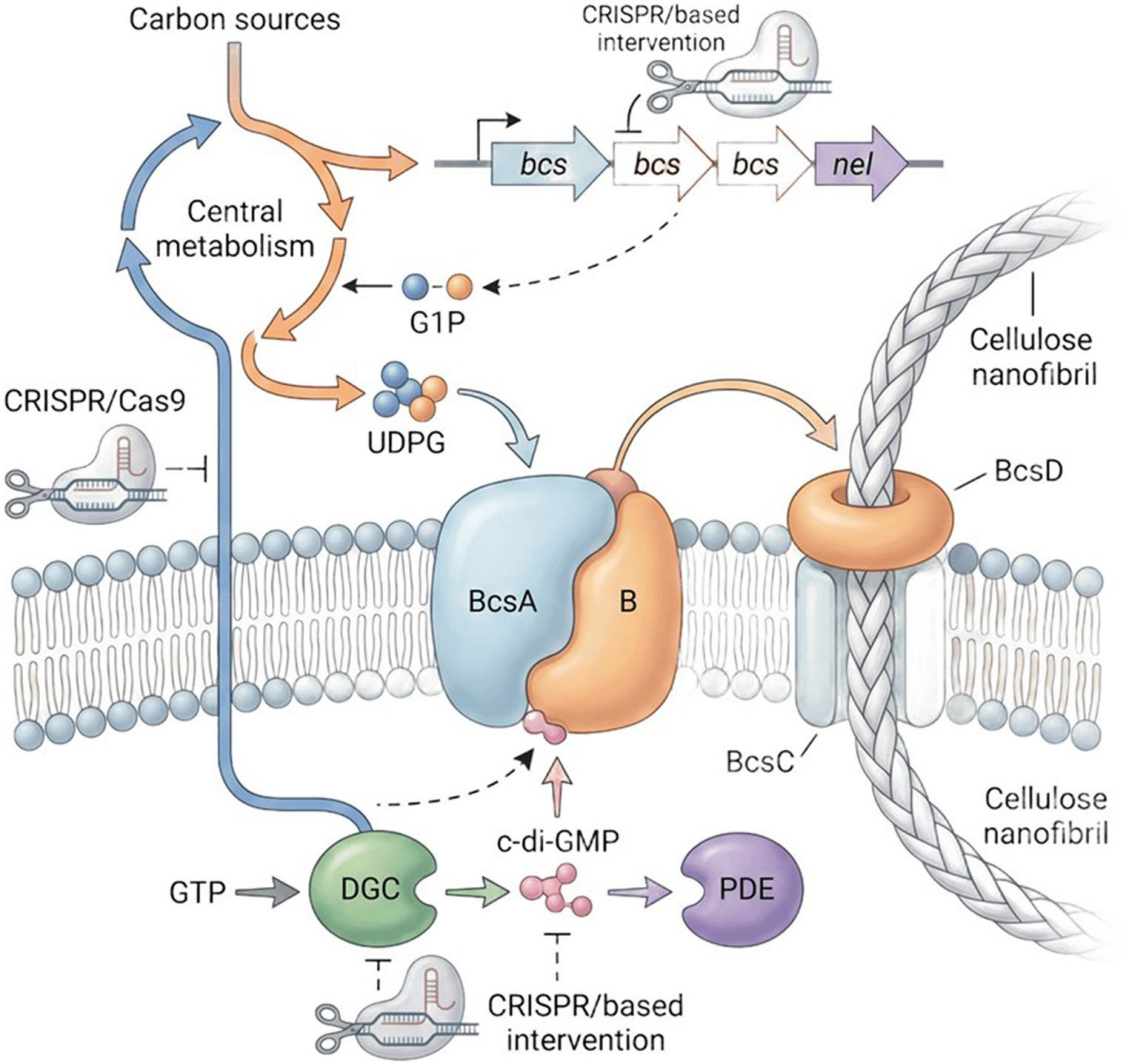
Schematic overview of bacterial cellulose biosynthesis in *Komagataeibacter* and intervention points for CRISPR-based engineering. Carbon sources are channelled through central metabolism to form glucose-1-phosphate and UDP-glucose, which feeds the BcsA–B cellulose synthase complex. Cellulose production is activated by c-di-GMP, whose intracellular levels are regulated by diguanylate cyclases and phosphodiesterases. Accessory proteins such as BcsC and BcsD assist polymer extrusion and nanofibril assembly. CRISPR-based tools can be used to edit or regulate genes involved in the *bcs* operon, c-di-GMP turnover and carbon flux, thereby modulating bacterial cellulose production and properties

BC is predominantly produced by members of the genus *Komagataeibacter* (e.g., *K. xylinus*, *K. hansenii* and *K. rhaeticus*) and is distinguished by its ability to generate a highly crystalline nanofibrillar network through the polymerisation of β-1,4-glucan chains mediated by the cellulose synthase machinery encoded by the *bcs* operon. In these bacteria, BC biosynthesis is initiated by the conversion of carbon substrates into UDP-glucose, the immediate precursor for glucan chain elongation. The catalytic core of the synthase complex is formed chiefly by BcsA and BcsB, whereas accessory components such as BcsC and BcsD are involved in polymer translocation, extracellular assembly and fibril organisation. A central layer of regulation is provided by cyclic di-GMP (c-di-GMP), a second messenger whose intracellular concentration is closely linked to activation of the biosynthetic apparatus and to the accumulation of cellulose nanofibrils (Fig. 1) (Huang et al. 2025). By allosterically stimulating the cellulose synthase complex, c-di-GMP functionally connects intracellular signalling with polymer output, while its turnover is governed by the antagonistic activities of diguanylate cyclases and phosphodiesterases.

Despite substantial progress in elucidating the structural and regulatory basis of BC formation, important questions remain regarding the integration of signalling networks with central carbon metabolism under distinct cultivation regimes, particularly in view of the need to improve productivity and process robustness at industrial scale. Within this framework, CRISPR-based strategies have emerged as promising tools for the rational engineering of *Komagataeibacter*, enabling targeted modification or transcriptional regulation of genes associated with cellulose biosynthesis, c-di-GMP homeostasis and carbon partitioning towards UDP-glucose. Such approaches expand the possibilities for fine control over BC titre, fibril ultrastructure and the resulting material properties, thereby strengthening the biotechnological relevance of this microbial platform.

This is evidenced by genetic engineering efforts employing modern tools such as CRISPR/Cas9 to modify genes within the BC synthase pathway, resulting in productivity increases of approximately 23.6%, thereby illustrating the importance of metabolic engineering strategies to overcome the natural physiological limitations of the producing bacterium (Huang et al. 2025).

Other studies have demonstrated that co-cultivation strategies can dramatically influence BC biosynthesis. For instance, the co-cultivation of *Bacillus cereus* with *K. xylinus* increased BC titre — from 1.2 to 4.4 g/L in enzymatic hydrolysate derived from lignocellulosic waste — an effect attributed to metabolites such as acetoin and 2,3-butanediol, which enhance cellular energy metabolism and the activity of enzymes associated with the synthesis process (Li et al. 2023).

Despite these advances, many of the strategies described to date do not systematically address production robustness at larger scales or the genetic stability of the strains — factors that continue to limit the transition of BC from laboratory studies to robust industrial applications.

### Structure, properties and modifications

BC is distinguished by its structural purity, as it is produced without lignin or hemicellulose, and by its highly organized nanofibrillar network. This structure results in high crystallinity and a remarkable capacity to retain water, features that contribute to its mechanical stability and biocompatibility. Such characteristics have made BC an attractive platform for applications in tissue engineering, biomedical devices, functional membranes, and active packaging (Pandey et al. 2024). In tissue engineering, for example, the suitability of BC-based scaffolds depends not only on intrinsic mechanical resistance but also on structural parameters such as pore size, porosity, and network architecture, which must be tailored according to the target tissue. While higher porosity may improve cell infiltration, nutrient diffusion, and overall scaffold lightness, it may simultaneously reduce mechanical strength, highlighting the need to balance structural integrity with biological functionality during material design.

Its fibrillar structure is highly amenable to modification, enabling the incorporation of nanomaterials or biopolymers and opening perspectives for bioactive materials with tailored properties. The high density of hydroxyl groups within the BC network favours chemical functionalisation through esterification, oxidation, or covalent bonding with bioactive agents, including proteins, polysaccharides, and functional nanoparticles. However, many approaches have yet to systematically investigate the effects of such modifications on biocompatibility and performance under real application conditions.

### Sustainable production and alternative substrates

One of the major challenges facing BC production is cost reduction, as it traditionally relies on carbon- and nitrogen-rich media such as standard Hestrin–Schramm medium. Recent research has therefore focused on intensifying BC production through the substitution of conventional substrates with agro-industrial residues and organic by-products. For example: (1) The use of fig waste as a carbon source for *K. xylinus* resulted in up to approximately 8.45 g/L of BC under optimised conditions — one of the highest titres reported to date for BC produced from alternative substrates (Yilmaz and Goksungur 2024). (2) The use of vinasse and *Aloe vera* in enriched culture media demonstrated that bioactive components can be incorporated into the BC network without compromising mechanical properties, while also adding value to the final product (Borro et al. 2023). (3) Studies further reveal that alternative nitrogen sources derived from agro-based flours (soybean, triticale, teff, quinoa) can positively influence BC production and fibril morphology, highlighting the importance of integrated medium optimisation strategies that consider not only carbon sources, but also nitrogen and micronutrients (Absharina et al. 2026).

Despite the progress observed, variability in the physicochemical properties resulting from different substrates still poses challenges for standardisation, particularly in applications that require consistent performance at large scale.

Although BC biosynthesis is biologically well characterised, recent literature — particularly experimental studies published in recent years — indicates that conventional strategies still lack integrated solutions to challenges such as high production costs, variability in material properties, and the genetic stability of the microbial producer in continuous or large-scale processes. The adoption of metabolic engineering and synthetic biology tools has emerged as a promising pathway, as illustrated by the use of CRISPR/Cas9 to enhance yields and modulate structural characteristics. However, there remains a need for studies evaluating performance in concrete applications (medical, food, packaging) under real industrial conditions, as well as life cycle assessments capable of demonstrating environmental benefits at technological scale.

Although BC is often regarded as an environmentally favourable cellulose platform, its advantages over plant-derived cellulose, cellulose derivatives and other bioplastics are not inherent; rather, they depend on how the material is produced, processed and managed across the life cycle (Foroughi et al. 2021; Ali et al., 2023). BC has an important theoretical environmental advantage because it is biosynthesised by fermentation as a highly pure material, thereby avoiding the lignin removal, pulping and bleaching stages typically required for plant cellulose. This may reduce dependence on harsh delignification chemistry and simplify downstream purification. In addition, BC can be produced from agro-industrial residues and side streams, which may improve circularity and reduce the use of virgin lignocellulosic feedstocks (Forte et al. 2021; Silva et al. 2025). However, these potential benefits can be offset by the burdens associated with culture medium preparation, energy demand, water use, low production titres, washing steps and especially drying and downstream processing, making life cycle assessment (LCA) essential for determining whether BC actually provides environmental gains at technological scale (Forte et al. 2021; Silva et al. 2025).

The first dedicated LCA of BC production, reported by Forte et al. (2021), demonstrated that BC should not automatically be considered a low-impact material simply because it is bio-based. Their cradle-to-gate assessment identified culture medium formulation and energy consumption as major contributors across several impact categories, highlighting the importance of process efficiency and upstream inputs in BC production. More recently, Silva et al. (2025) expanded this discussion by comparing BC with other cellulosic sources and showed that the environmental profile of BC is highly route-dependent. In that study, BC pulp displayed lower environmental impacts than nanocelluloses, whereas BC-lyocell exhibited higher impacts than cotton, viscose and lyocell in most categories, except for land use and water depletion. These findings are particularly relevant because they move beyond generic sustainability claims and demonstrate that the environmental competitiveness of BC is strongly influenced by culture medium composition, washing requirements, NaOH use and energy inputs (Forte et al. 2021; Silva et al. 2025).

In comparison with plant cellulose and some cellulose derivatives, BC offers the advantage of direct microbial synthesis into a nanostructured and chemically pure material, without the extraction and bleaching stages associated with wood- or plant-based cellulose. Nevertheless, plant-derived celluloses benefit from mature industrial infrastructure and large-scale supply chains, whereas some cellulose derivatives may carry substantial burdens from chemical modification. For example, Liu et al. (2024) reported that a cellulose acetate-based composite showed higher environmental impacts than a conventional PP–glass fibre reference across all assessed categories, with acetic anhydride production identified as the main hotspot. More broadly, Foroughi et al. (2021) emphasised that cellulose-based materials cannot be assumed to be uniformly low-carbon, since their impacts depend strongly on feedstock origin, processing chemistry, solvent recovery, energy source and end-of-life scenario. Thus, BC may outperform chemically derivatised celluloses in selected applications, especially when produced from waste-derived media and subjected to mild downstream processing, but such an advantage must be demonstrated on a case-by-case basis through robust LCA.

When compared with other bioplastics such as PLA, PHB and cellulose-based thermoplastics, BC remains promising because it is bio-based, biodegradable and can function directly as a film, membrane or hydrogel without requiring thermoplastic derivatisation. Even so, the wider LCA literature on bioplastics consistently shows that environmental superiority is not guaranteed and depends on feedstock selection, production route, energy demand and end-of-life management (Ali et al., 2023). Senila et al. (2024), for instance, performed an LCA of PLA and PHB production from lignocellulosic waste and reinforced the view that bio-based origin alone is insufficient to ensure lower environmental impact, since each system presents distinct burdens across categories and technological stages. Consequently, any claim that BC is environmentally preferable to other bio-based materials should be supported by comparative LCAs that include not only renewable origin and biodegradability, but also realistic assumptions regarding service life, scale-up, processing intensity and disposal pathways.

## Functional engineering of bacterial cellulose-based biocomposites

### Chemical and physical modification

The chemical functionalisation of bacterial cellulose has evolved beyond simple transformations, aiming to impart specific surface properties and mechanical performance that meet functional requirements in biomedical and smart material applications. Strategies widely explored in the literature include TEMPO-mediated oxidation (2,2,6,6-tetramethylpiperidine-1-oxyl) to generate reactive carboxyl groups, as well as esterification reactions with multifunctional agents to introduce hydrophobic or bioactive moieties (Rahmadiawan et al. 2024).

For example, TEMPO-oxidised BC subsequently conjugated with bioactive peptides has been shown to promote cell adhesion and osteogenic differentiation in scaffolds for bone regeneration without compromising biocompatibility (Liu et al. 2025). The introduction of hydrophobic groups via esterification has also been employed to enhance structural stability in humid environments, a critical requirement for implantable devices. However, such modifications may reduce the intrinsic water-retention capacity of BC and alter its biodegradability, thereby introducing functional trade-offs that still lack systematic investigation (Jiang et al. 2023).

**Fig. 2:**
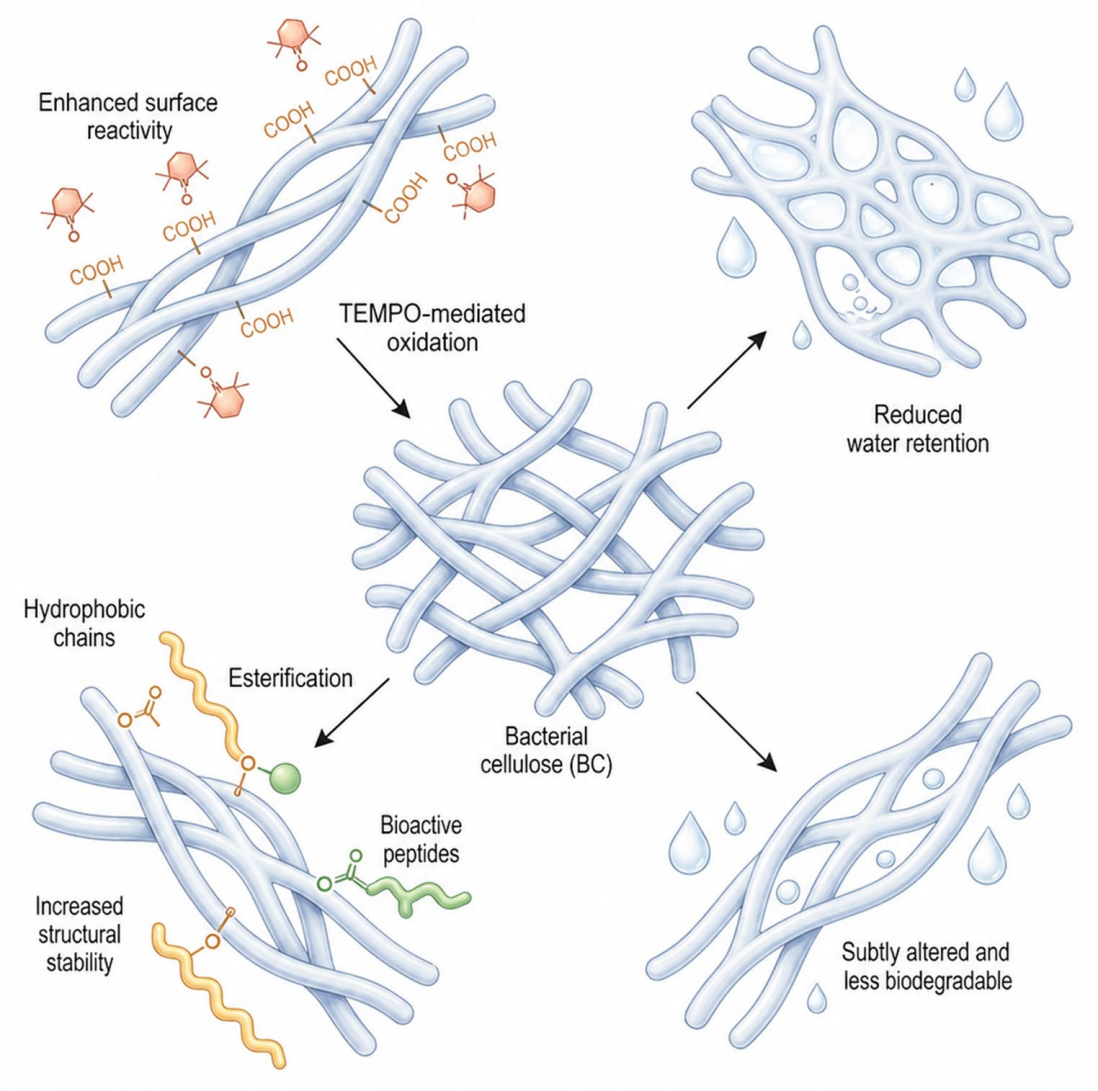
Chemical functionalisation strategies of bacterial cellulose to tailor surface properties and mechanical performance for biomedical and smart material applications Image creation was assisted using the online illustration platform Illustrae (https://illustrae.co/)

Figure 2 presents the main chemical functionalisation strategies for bacterial cellulose, highlighting how these approaches enable modulation of surface properties and mechanical performance for biomedical and smart material applications.

To provide a structured overview of the main chemical strategies employed to tailor the properties of bacterial cellulose, Table 1 summarises representative modification routes, including oxidation, esterification, and advanced click-chemistry approaches, along with their respective reagents, targeted property changes, and validated applications. The table highlights how specific chemical transformations directly influence surface functionality, mechanical performance, and bioactivity, thereby enabling the adaptation of bacterial cellulose to diverse biomedical and smart material requirements. Collectively, these examples illustrate current trends in chemical functionalisation and underscore the inherent trade-offs between enhanced functionality and the preservation of intrinsic properties, such as water retention and biodegradability.

**Table 1:** Chemical modification strategies of bacterial cellulose and their effects on functional properties and applications

Type of Modification	Agent/Reagent	Modified Property	Application Tested	Reported Effect	Reference
TEMPO oxidation + peptide conjugation	TEMPO + peptide	Bioactivity and cell adhesion	Bone tissue engineering	↑ Osteogenesis in vitro	Liu et al. 2025
Esterification with hydrophobic agents	Aliphatic anhydrides	Hydrophobicity	Implantable devices	↑ Stability in humid environments	Jiang et al. 2024
Photo-activated click chemistry	Click reagents + UV light	Site-specific functional modification	Smart materials	Greater spatial control	Fan et al. 2022; Li et al. 2024

An additional criticism concerns the need for spatial control over chemical modifications, as non-selective reactions may lead to structural heterogeneity at nano- and microscopic scales, thereby adversely affecting mechanical properties and mass transport behaviour. Recent studies applying light-controlled click chemistry illustrate promising approaches to overcome these limitations; however, long-term evaluations of functional stability and inflammatory response in complex biological models remain lacking.

From a biomedical engineering perspective, an aspect that remains insufficiently explored in many studies on BC concerns the material’s ability to be hygienised and sterilised without compromising its structural and functional properties, a fundamental requirement for in vivo experiments and eventual clinical application. Although the literature widely highlights the biocompatibility, high water-holding capacity, and nanofibrillar structure of BC, discussion regarding sterilisation protocols and their effects on material performance is still relatively limited. This issue is particularly relevant because biomaterials intended for advanced wound dressings, implants, or tissue engineering scaffolds must comply with strict requirements of sterility and biological safety.

Recent studies indicate that methods such as gamma irradiation and electron beam irradiation can be applied to sterilise BC-based materials while preserving key properties required for biomedical applications. For instance, Tranquilan-Aranilla et al. (2024) demonstrated that irradiation doses between 15 and 25 kGy, a range typically employed for the sterilisation of medical devices, preserve the functional integrity of BC-based wound dressings, maintaining important properties such as exudate absorption capacity and water vapour permeability. Furthermore, investigations into the modification and processing of BC indicate that different physical and chemical treatments may influence parameters such as crystallinity, fibrillar morphology, and mechanical strength, highlighting the need to carefully evaluate the effects of sterilisation processes during the development of BC-based biomaterials (Wang et al. 2024). Therefore, the systematic inclusion of studies addressing post-sterilisation structural stability, biological compatibility, and functional performance represents a crucial step towards translating bacterial cellulose from laboratory-scale research to practical biomedical applications and medical devices.

## Nanocomposites and hybrid materials

The incorporation of nanofillers into the BC matrix has considerably broadened the functional potential of this biomaterial, particularly in regenerative medicine, wound healing and bone tissue engineering. In these systems, nanofillers do not act solely as reinforcing agents, but also influence pore architecture, surface roughness, mineralisation behaviour, mechanical performance and cell–material interactions. As a result, BC-based composites can overcome some of the intrinsic limitations of pristine BC, such as its low bioactivity in hard-tissue applications and its lack of antimicrobial or electrically active functionality. Recent reviews have highlighted that these improvements are strongly dependent on the homogeneous dispersion of the nanophase within the three-dimensional fibrillar network and on the maintenance of the high porosity and water-holding capacity that characterise BC (Bacakova et al. 2023).

Among carbon-based nanofillers, graphene oxide (GO) is one of the most extensively investigated because of its ability to enhance stiffness, interfacial interactions and biological signalling, while also introducing conductive and antimicrobial properties. Recent studies and reviews have shown that GO-containing cellulose composites often display improved physicochemical performance together with enhanced cell adhesion and proliferation, especially in wound healing and scaffold applications (Salehi et al. 2023). However, these benefits are concentration-dependent, and biosafety remains a key concern for translational use. Experimental evidence indicates that GO concentrations above approximately 50 µg/mL may induce stronger cytotoxic, inflammatory or genotoxic responses in mammalian cells, whereas lower concentrations are generally better tolerated, depending on flake size, surface chemistry, exposure time and cell type (Teimoorian et al. 2023). Therefore, any discussion of BC/GO systems should include not only their mechanical and biological advantages but also their dose-dependent safety limitations.

Another important class of hybrid systems is based on the incorporation of hydroxyapatite (HAp), which is particularly relevant for bone-related applications because it introduces osteoconductive properties absent in neat BC. Recent work has shown that HAp-containing BC scaffolds can combine a mineral phase favourable for bone regeneration with the highly interconnected fibrous structure of BC, which supports cell infiltration and nutrient transport. For example, bacterial nanocellulose/chitosan/gelatin/HAp scaffolds have been reported with surface pore diameters ranging from 384.5 to 457.4 μm and internal pore diameters from 467.5 to 498.7 μm, together with porosity values between 66.0 and 81.4%. In the same study, the incorporation of 0.1–0.2% (w/v) HAp improved compressive strength, thermal stability and antibacterial performance, indicating that low mineral loadings may already be sufficient to enhance scaffold functionality (Phatchayawat et al. 2025). These values are particularly relevant because pore sizes above 300 μm are generally considered favourable for bone ingrowth and vascularisation.

More sophisticated ternary systems combining BC, HAp and GO have also shown considerable promise, since they integrate the fibrillar template of BC, the osteoconductivity of HAp and the reinforcing and interfacial contributions of GO. Challa et al. (2025) reported that a BC/HAp/GO scaffold reached a compressive strength of approximately 13.4 MPa, while also supporting fibroblast proliferation and the expression of osteogenesis-related markers. Although this value is still below that of dense cortical bone, it is relevant for several non-load-bearing and moderately loaded tissue-engineering applications, and clearly demonstrates the reinforcing effect of hybridisation compared with unmodified BC matrices. Nevertheless, mineralised BC systems still present important challenges, including possible mineral phase disaggregation under repeated loading and the lack of long-term functional validation in vivo, which limits direct clinical translation (Bayir et al. 2019; Challa et al. 2025).

Overall, the current literature demonstrates that BC-based nanocomposites can provide measurable improvements in mechanical, structural and biological performance, but the magnitude and relevance of these gains are highly dependent on composition, nanofiller concentration and target application. GO-containing systems are particularly attractive for reinforced and biologically responsive soft scaffolds, although their toxicological profile must be carefully controlled. HAp-containing systems are more directly suited to bone repair because of their improved mineralisation behaviour, roughness and osteoconductive potential, with recent formulations reporting porosity values of 66–81% and pore diameters approaching 500 μm. Ternary BC/HAp/GO systems appear to offer the most balanced multifunctionality, with compressive strengths around 13 MPa, but most of the currently available evidence remains based on proof-of-concept studies and short-term biological assays. Accordingly, future studies should place greater emphasis on quantitative comparisons of porosity, compressive strength, cell viability, mineralisation response and long-term biosafety in order to strengthen the translational relevance of these hybrid materials.

To illustrate representative examples of bacterial cellulose (BC)-based nanocomposites and the properties they enhance for specific target applications, Table 2 summarises key nanofiller systems, the performance improvements they confer, associated application domains, and remaining challenges for functional translation. (Table 3)

**Table 2:** Representative bacterial cellulose-based nanocomposites with enhanced mechanical or bioactive performance and their target applications

Nanocomposite System	Nanofiller	Property Enhancement	Target Application	Remaining Challenges	Reference
BC + Graphene Oxide (GO)	GO	↑ Mechanical strength	3D scaffolds, biosensors	Toxicity at high concentrations	Challa et al. 2025
BC + Hydroxyapatite (HAp)	HAp	↑ Stiffness and bone bioactivity	Bone tissue engineering	Long-term mechanical stability	Phatchayawat et al. 2025
BC + Nanocrystalline Cellulose (NCC)	NCC	↑ Elastic modulus	Active packaging	Interfacial compatibilisation	Rollini et al. 2020; Silva et al. 2023

**Table 3:** Complementary mechanical roles of bacterial cellulose, fungal mycelium, and hybrid systems in biofabricated biocomposites

Component	Primary Contribution	Mechanism of Action	References
Bacterial cellulose (BC)	Increases stiffness and tensile strength	Continuous nanofibrillar network acting as tensile reinforcement and forming bridges between matrix elements	Elsacker et al. 2021; Hoenerloh et al. 2022
Mycelium	Compressive structure and biological binding	Organic matrix that fills and binds particles; growth integrates biological materials	Kaffash et al. 2026
BC–mycelium hybrid	Improved internal bonding and tunable properties	Integration of mycelium within the BC network, increasing contact area and interfacial adhesion	Piórecka et al. 2023; Kaffash et al. 2026

**Fig. 3:**
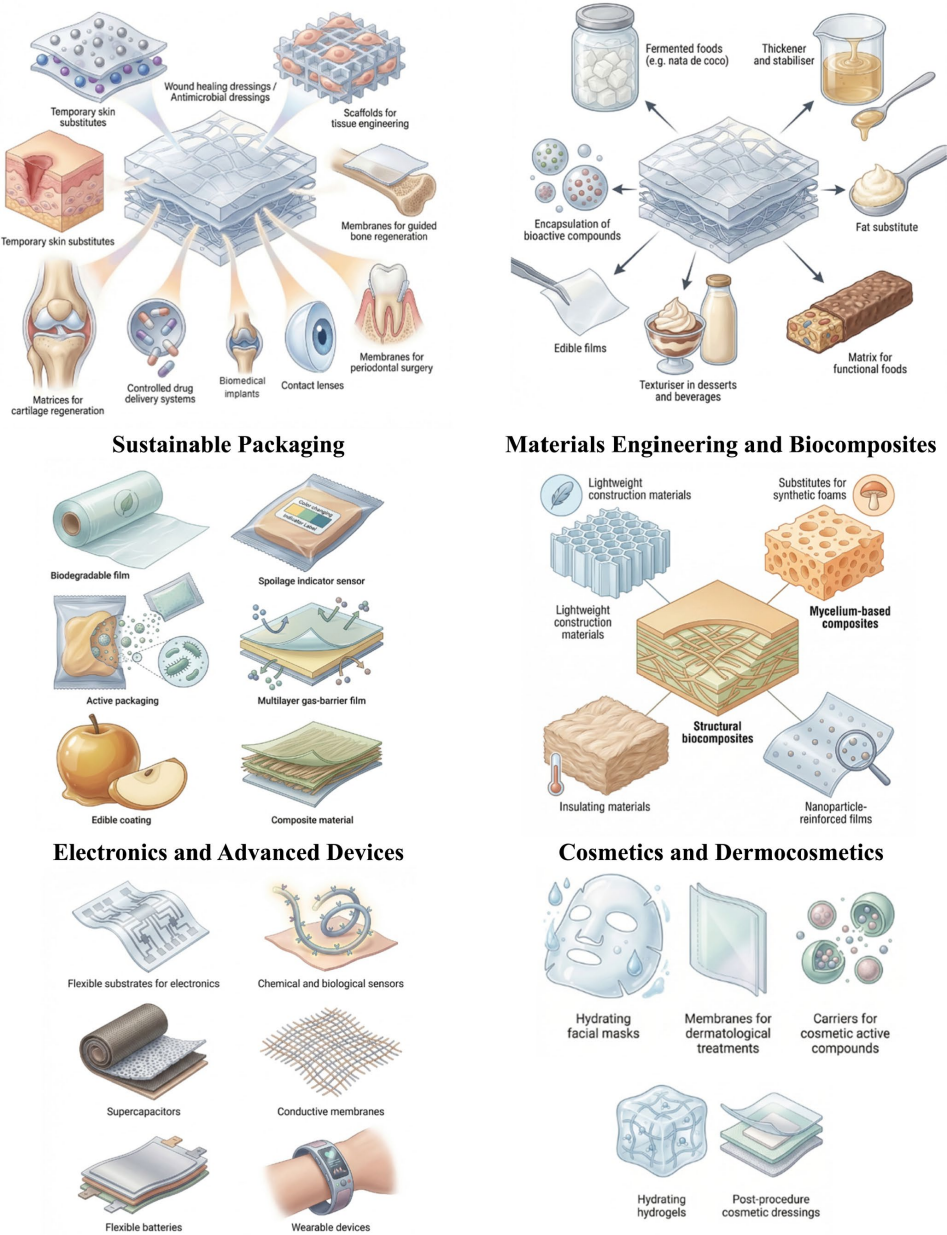
Schematic representation of emerging and advanced application sectors of bacterial cellulose, including filtration and water treatment, textile and sustainable fashion industries, agriculture, and biofabrication of living materials. Image creation was assisted using the online illustration platform Illustrae (https://illustrae.co/)

**Fig. 4:**
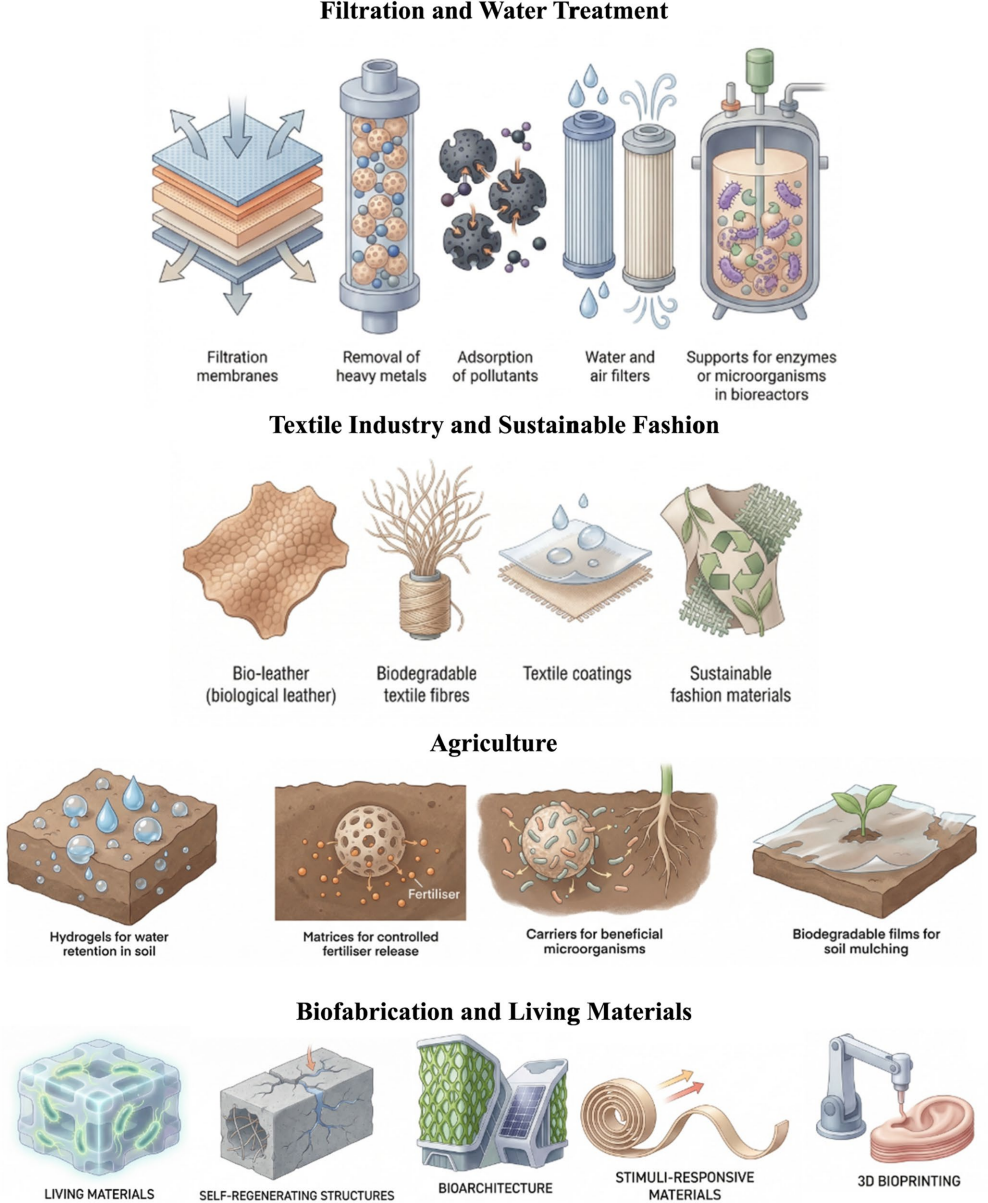
Schematic representation of the diverse application sectors of bacterial cellulose across biomedical, food, packaging, materials engineering, electronics, and cosmetic industries. Image creation was assisted using the online illustration platform Illustrae (https://illustrae.co/)

The wide range of applications of bacterial cellulose enables this material to be utilised across multiple sectors, reflecting its remarkable versatility and functional properties. Owing to its biocompatibility, mechanical strength and high purity, bacterial cellulose can be applied in diverse fields, supporting a broad spectrum of technological and industrial uses, as illustrated in Figs. 3 and 4.

## Biofabrication and advanced 3D structures

The integration of BC with advanced biofabrication methods has enabled the production of three-dimensional structures with improved control over micro- and macro-architecture, a requirement that is particularly important for tissue engineering and regenerative medicine. BC can be processed as hydrogels or incorporated into bioinks, supporting gel-assisted 3D printing and related additive manufacturing approaches in which pore organisation, interconnectivity and orientation can be rationally modulated (Utoiu et al. 2024). In this context, printed BC-based scaffolds may better mimic aspects of natural tissue architecture, favouring cell adhesion, migration, extracellular matrix deposition and nutrient exchange.

A key advantage of 3D printing is the possibility of overcoming one of the main limitations of conventional BC scaffolds: the difficulty of tailoring internal geometry without compromising the intrinsic nanofibrillar network. For example, BC-based scaffolds combined with polymeric matrices such as polylactide have shown improved cell adhesion and proliferation when printed with oriented porous structures, indicating that macrostructural control can directly influence biological response (Wu et al. 2022). Similarly, studies on scaffolds with interconnected pores reinforce that a well-defined porous architecture facilitates cell infiltration and nutrient transport, both of which are essential for functional cell viability (Bayir et al. 2019).

Nevertheless, the main challenge is not simply printing BC-containing formulations, but preserving hierarchical continuity across scales. The printed meta-structure must be integrated with the intrinsic nanofibrillar network of BC; otherwise, local heterogeneity, weak interfaces and uneven pore collapse may compromise mechanical performance, particularly when structures are scaled to larger volumes. In addition, the crystallinity of BC and its dependence on oxygen diffusion during biosynthesis still impose constraints on the fabrication of complex geometries and uniform internal architectures (Wu et al. 2022; Utoiu et al. 2024).

The incorporation of living cells, organoids or microbial components into BC-based printed systems further expands their potential, but also introduces additional design requirements. Long-term viability depends on the simultaneous control of hydration, nutrient diffusion, oxygen transport, mechanical stress and degradation behaviour under simulated physiological conditions. Although BC is widely recognised as a biocompatible scaffold for tissue engineering, limitations in structural customisation, large-scale fabrication and prolonged biological validation still restrict broader biomedical implementation (Pasaribu et al. 2024; Pandey et al. 2024).

Future progress in BC-based biofabrication will therefore require integrative strategies that balance printability, mechanical robustness, hierarchical architecture and biological performance. Rather than treating 3D printing as an independent fabrication step, it should be combined with material engineering and biological control to generate reproducible structures with predictable porosity, nutrient transport, cell viability and mechanical stability. Such advances are essential to move BC-based systems from proof-of-concept models towards functional biomaterials for tissue engineering, regenerative medicine and programmable living-material platforms.

### Fungal mycelium-based biocomposites

#### Growth, properties and substrate influence

Recent studies show that the performance of mycelium-based composites (MBCs) is governed less by the mere presence of fungal biomass and more by the interaction between fungal species, substrate architecture and processing strategy. In practice, the most relevant design challenge is not whether mycelium can bind lignocellulosic particles, which is already well established, but how this biological binding can be controlled to achieve reproducible combinations of density, porosity, strength and insulation performance suitable for specific end uses (Li et al. 2025; Pertile et al. 2025).

A recurring trade-off in the literature concerns porosity versus mechanical integrity. Higher porosity is generally associated with lower density and improved thermal or acoustic insulation, but this typically occurs at the expense of mechanical resistance because the solid fraction and continuity of the load-bearing network are reduced. This relationship explains why MBCs are often more successful in non-structural or semi-structural applications than in load-bearing uses. Accordingly, recent work has increasingly focused on identifying substrate combinations and cultivation conditions that mitigate this compromise rather than simply maximising fungal colonisation (Li et al. 2025; Fritz et al. 2025).

Substrate selection is one of the most influential variables in this optimisation process. Fritz et al. (2025) showed that composites produced from mixed agroforestry residues, particularly hazelnut shells combined with pine sawdust, outperformed materials derived from a single vegetal residue, exhibiting higher modulus of rupture and internal bond strength. These results are important because they indicate that performance gains arise not only from chemical composition, but also from the structural heterogeneity created by combining particles of different morphology and rigidity. Such heterogeneity appears to favour more effective mycelial interlocking, improved particle cohesion and better stress distribution throughout the composite. Rather than treating the substrate merely as a nutrient source, these findings support viewing it as a structural design variable that directly shapes the final engineering properties of the material.

A similar trend was reported by Jiménez-Obando et al. (2025), who demonstrated that *Ganoderma lucidum* cultivated on composite substrates based on natural fibres and plant residues generated materials with low density and reduced thermal conductivity. These findings reinforce the view that substrate formulation can be used strategically to tailor MBCs for insulation-oriented applications. However, the same studies also suggest that improvements in thermal performance are frequently associated with increased porosity, again raising the issue of reduced strength and dimensional robustness. Thus, claims regarding the multifunctionality of MBCs should be framed with greater caution, since not all favourable properties can be maximised simultaneously.

Processing conditions introduce an additional layer of variability that is still insufficiently standardised across studies. Li et al. (2025) and Pertile et al. (2025) emphasised that industrially relevant production routes capable of preserving the three-dimensional organisation of the initial substrate during fungal colonisation may retain low density and high porosity while still achieving compressive strength values around 0.48 MPa, which is comparable to some lightweight construction materials. Although such results are promising, they also highlight an important limitation of the field: similar nominal formulations may yield substantially different properties depending on particle size distribution, packing, moisture content, growth time, pressing conditions and drying regime. This lack of process harmonisation remains a major obstacle to reproducibility and scale-up.

Taken together, recent evidence suggests that advances in MBC development will depend less on reiterating the general sustainability of mycelium and more on establishing clear structure–property–process relationships. The most relevant progress has come from studies that treat fungal species, substrate composition and manufacturing parameters as interacting design factors rather than isolated variables. From this perspective, the future of MBCs lies not simply in their biodegradability or bio-based origin, but in the ability to engineer these materials with predictable performance, reduced batch-to-batch variability and stability under realistic service conditions. These issues remain central if MBCs are to move beyond niche insulation and packaging applications towards broader industrial implementation.

**Fig. 5:**
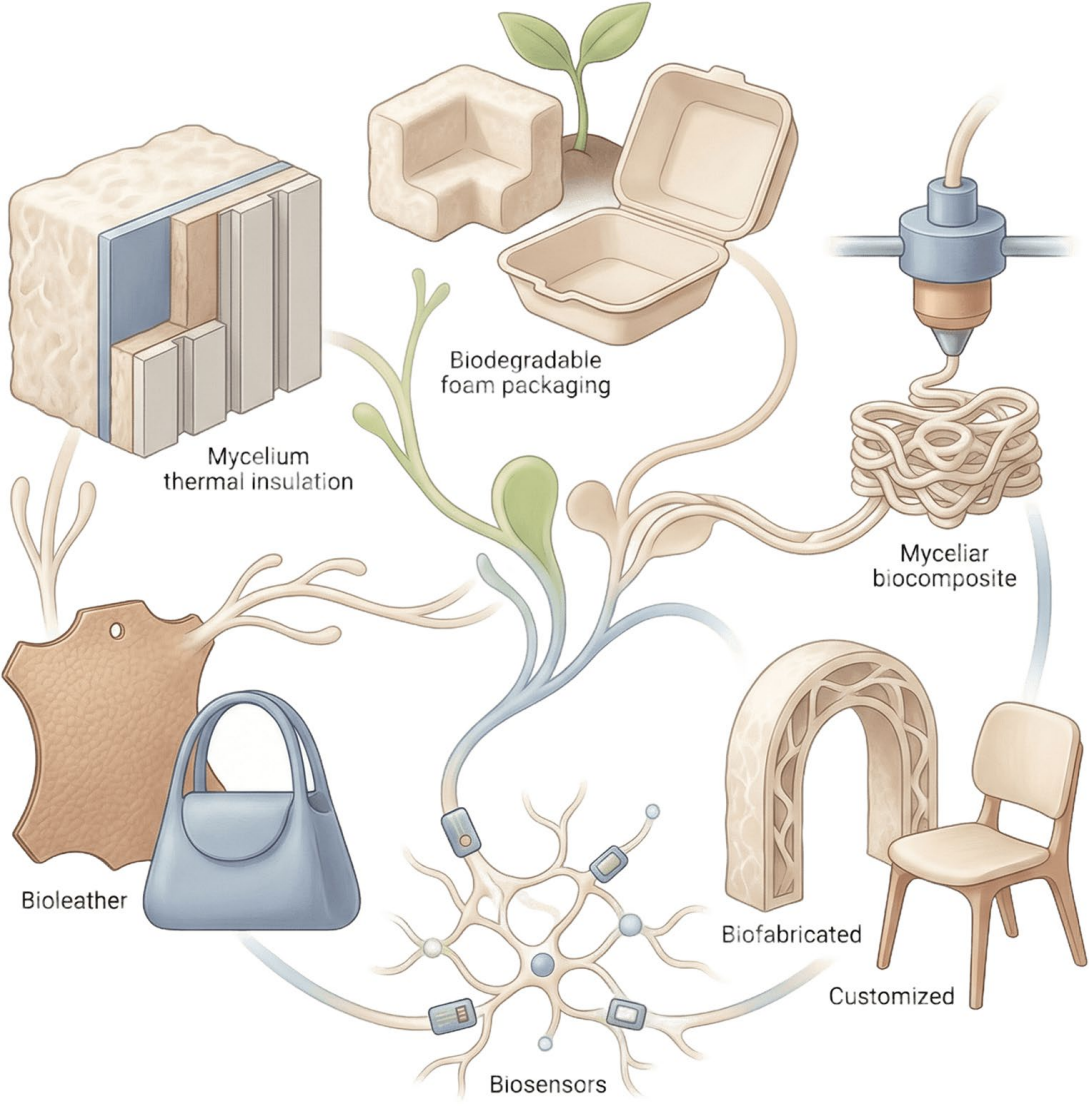
Schematic representation of the main industrial sectors in which fungal mycelium-based biocomposites are applied

Figure 5 summarises representative sectors in which fungal mycelium-based biocomposites are currently being explored, with emphasis on packaging, construction, textiles and other bio-based product applications.

## Development of mycelium composites and functionality

Literature published after 2020 indicates that mycelium-based composites (MBCs) represent an emerging class of multifunctional biomaterials, with applications ranging from biodegradable packaging, lightweight construction materials, and thermal and acoustic insulation to sustainable design and biofabricated flexible materials. This broad applicability arises from the ability of fungal mycelium to form a continuous hyphal network that acts simultaneously as a structural matrix and binding agent, consolidating low-cost and widely available lignocellulosic substrates such as agricultural and forestry residues (Elsacker et al. 2021; Jones et al. 2020).

Recent advances demonstrate that cultivation process engineering — including the selection of fungal species, substrate composition and granulometry, growth time, and inactivation methods — allows significant modulation of properties such as apparent density, porosity, compressive strength, and thermal conductivity. Experimental studies indicate that MBCs can achieve mechanical performance comparable to low-density synthetic materials, while simultaneously maintaining complete biodegradability and reduced environmental impact (Elsacker et al. 2021; Li et al. 2025).

In addition, recent work highlights the development of flexible mycomaterials obtained through controlled mycelial growth on refined lignocellulosic substrates, resulting in sheet-like structures with mechanical behaviour comparable to soft natural materials. These biofabricated systems have been proposed primarily as sustainable alternatives to leather, rather than conventional woven textiles, since they form continuous non-woven structures produced by fungal networks. Studies have shown that such materials can combine flexibility, moderate mechanical strength, and tunable surface properties, while allowing post-processing treatments such as pressing, coating, or finishing to improve durability and aesthetic characteristics (Jones et al. 2021; Khan 2025). By avoiding petrochemical polymers and relying on fungal self-assembly, these mycelium-derived materials expand the functional potential of mycelium-based composites (MBCs) for applications in fashion, design, and other technical products.

### Hybrid composites and mechanical enhancements

To improve the BC–mycelium relationship, the section should move from a descriptive list of fabrication routes to a more mechanistic discussion centred on interface engineering, growth compatibility and hierarchical load transfer. Recent work on mycelium composites and living-material fabrication suggests that the performance of BC–mycelium hybrids depends less on simply combining the two phases and more on controlling four variables: (i) BC surface chemistry and hydration, (ii) fungal adhesion and penetration into the BC network, (iii) spatial distribution of the two phases, and (iv) post-growth densification and drying (Hoenerloh et al. 2022; Camilleri et al. 2025). In other words, the most effective “symbiosis” is achieved when BC is not treated merely as an inert reinforcement, but as a bioactive template that supports hyphal attachment, guides colonisation and improves interfacial cohesion across scales (Hoenerloh et al. 2022; Piórecka et al. 2023).

A stronger version of this section could therefore state that the BC–mycelium interaction may be improved by tailoring BC morphology and accessibility before fungal inoculation. Highly hydrated and porous BC networks favour fungal attachment, but excessive compaction or poor pore interconnectivity may restrict hyphal penetration and lead to surface-limited colonisation. Conversely, partial disintegration of BC into nanofibres or the use of loosely assembled BC mats can increase the available contact area, enhance mechanical interlocking with hyphae and improve stress transfer within the hybrid matrix. This is especially important because the principal reinforcement mechanism is not only the intrinsic tensile strength of BC, but the formation of a multiscale network in which BC nanofibrils bridge microvoids and fungal hyphae provide larger-scale binding and structural continuity (Elsacker et al. 2021; Hoenerloh et al. 2022).

The compatibility between the two biological systems can also be enhanced by matching cultivation conditions more carefully. Sequential growth is often more controllable than true co-cultivation because cellulose-producing bacteria and filamentous fungi differ markedly in oxygen demand, growth kinetics and nutrient use; if these differences are not balanced, one organism can dominate the system and compromise the architecture of the hybrid. A more effective strategy is often to produce BC first under conditions that optimise fibril formation, and only then inoculate the fungus under moisture and nutrient conditions that favour controlled penetration rather than superficial overgrowth. This approach improves reproducibility and allows the BC phase to act as a pre-designed scaffold instead of a passive by-product of a mixed culture (Hoenerloh et al. 2022; Piórecka et al. 2023).

Another important route to improving the BC–mycelium interface is surface and substrate engineering. Because fungal adhesion depends strongly on local topography, water activity and available anchoring points, hybrid performance can be enhanced when BC is combined with lignocellulosic particles or natural fibres that create a graded architecture rather than an abrupt BC-only/fungus-only boundary. In such systems, BC can improve fine-scale particle adhesion, while hyphae reinforce the broader substrate framework. This hierarchical arrangement is likely to reduce crack initiation, improve energy dissipation under compression and enhance dimensional stability. The literature on mycelium composites increasingly shows that mechanical performance depends heavily on how well fibres, particles and fungal biomass are integrated, rather than on fungal growth alone (Elsacker et al. 2021; Camilleri et al. 2025).

From a product-performance perspective, an improved BC–mycelium relationship can enhance final properties in at least four ways. First, it can increase stiffness and tensile integrity by improving interfacial adhesion and reducing weak points between particles. Second, it can improve compressive behaviour and energy absorption, because a better integrated hierarchical network distributes stress more efficiently across the material. Third, it can enhance dimensional stability and crack resistance during drying, since BC can reduce local shrinkage heterogeneity if it is well dispersed. Fourth, it may allow better control of anisotropy and functional gradients, especially if BC orientation is used to guide directional fungal growth and internal architecture. These benefits are highly plausible from a structure–property standpoint, but the field still needs more direct quantitative comparisons between poorly integrated and well-integrated hybrids to demonstrate how much each parameter contributes (Elsacker et al. 2021; Hoenerloh et al. 2022; Camilleri et al. 2025).

A more critical and review-appropriate conclusion for this subsection would therefore be that the key issue is not simply whether BC reinforces mycelium composites, but under which interfacial and processing conditions BC acts as an effective scaffold rather than an inert additive. Future work should focus on quantifying fungal penetration depth into BC, interfacial bonding quality, shrinkage behaviour, mechanical anisotropy and long-term stability under variable humidity. Framing the discussion in this way would make the section more analytical and would respond more directly to the question of how BC–mycelium integration can be improved and how this translates into superior end-product performance (Hoenerloh et al. 2022; Camilleri et al. 2025).

The main advantage of combining BC with mycelium does not lie merely in adding one bio-based phase to another, but in creating a hierarchically integrated network in which BC provides nanoscale reinforcement and fungal hyphae generate microscale binding and structural continuity (Elsacker et al. 2021; Hoenerloh et al. 2022). From this perspective, the central question is how to engineer the interface between these two living-derived phases so that adhesion, load transfer and architectural coherence are maximised. Current evidence suggests that hybrid performance depends strongly on BC morphology, hydration state, fungal penetration, phase distribution and post-growth processing rather than on the simple presence of both components (Hoenerloh et al. 2022; Camilleri et al. 2025).

In sequential systems, BC can serve as a pre-formed scaffold for fungal colonisation, provided that its structure remains sufficiently open to permit hyphal penetration. Highly compact BC layers may restrict fungal ingrowth and limit the interaction to surface attachment, whereas more porous or partially disintegrated BC networks increase contact area and improve mechanical interlocking. Under these conditions, BC is expected to bridge microstructural discontinuities, enhance particle-to-particle cohesion and reduce local stress concentration, while the fungal mycelium consolidates the larger-scale matrix. The result is a more efficient multiscale load-bearing architecture than that achieved by either phase alone (Elsacker et al. 2021; Piórecka et al. 2023).

Co-cultivation offers an appealing route towards living hybrid materials, but it remains difficult to control because cellulose-producing bacteria and filamentous fungi differ in oxygen requirements, metabolic rates and nutritional preferences. For this reason, the most effective hybrid designs currently appear to be those in which BC production and fungal colonisation are at least partially decoupled, allowing each phase to be formed under conditions favourable to its own structural role. This also improves reproducibility and facilitates the rational tuning of composite architecture (Hoenerloh et al. 2022).

The interface between BC and mycelium can be further improved by combining both phases with lignocellulosic particles or natural fibres that create a graded structural environment. In such systems, BC acts as a nanofibrillar binder that improves local adhesion and stress transfer, whereas hyphae reinforce the broader substrate network. This hierarchical organisation is likely to improve stiffness, compressive resistance, crack deflection and dimensional stability, while still preserving the low density and porosity required for lightweight applications (Elsacker et al. 2021; Camilleri et al. 2025).

Accordingly, the most promising route for improving BC–mycelium hybrids is to treat BC not as a passive additive but as a templating and interfacial phase capable of guiding fungal colonisation and reinforcing the developing network. Better control of BC openness, surface accessibility, moisture distribution and fungal growth kinetics should enhance interfacial cohesion and produce composites with more predictable mechanical behaviour. In final products, this improved integration may translate into higher stiffness, better energy absorption, lower shrinkage-induced damage and greater architectural control, including the possibility of designing anisotropic materials with application-specific performance (Hoenerloh et al. 2022; Piórecka et al. 2023; Camilleri et al. 2025).

Beyond the structural and functional advantages of combining BC and mycelium, the feasibility of products developed from such a consortium must also be considered from a manufacturing and application perspective. Although these hybrid systems are conceptually attractive because they integrate nanoscale reinforcement with biologically driven macroscopic binding, their practical implementation depends on several factors, including reproducibility of co-culture or sequential growth, process compatibility between microbial partners, scalability of production, drying behaviour, dimensional stability and long-term storage performance. In addition, the feasibility of the final product is closely linked to the intended application: for non-structural products such as packaging, insulation panels and lightweight design elements, BC–mycelium hybrids appear particularly promising because moderate mechanical performance may already be sufficient, while the bio-based origin and low-density structure offer clear advantages. In contrast, for applications requiring high structural reliability, moisture resistance or prolonged service life, the feasibility remains more limited and depends on improved process standardisation, better interface control and more robust validation under realistic environmental conditions. Thus, the practical viability of BC–mycelium consortia should not be assessed solely in terms of successful biological integration, but also in relation to manufacturability, cost, reproducibility and fitness for purpose in the target end use.

### Synthetic biology and engineered living materials

#### Synthetic biology in bacterial cellulose and mycelium-based materials

Synthetic biology has become an important enabling approach for the development of next-generation microbial biocomposites, particularly those based on bacterial cellulose (BC) and fungal mycelium. By combining genetic engineering, metabolic regulation and programmable biological circuits, synthetic biology allows microorganisms to be designed not only as producers of structural materials, but also as active agents capable of controlling material formation, functionality and responsiveness.

In BC-producing bacteria, especially species of the genus *Komagataeibacter*, synthetic biology tools have enabled significant advances in the modulation of cellulose biosynthesis, material morphogenesis and functional programming. Recent strategies include the engineering of the *bcs* operon, manipulation of c-di-GMP-regulated pathways and the insertion of synthetic genetic circuits capable of controlling the temporal and spatial deposition of cellulose nanofibrils (Zhang et al. 2021; Bimmer et al. 2022). These approaches make it possible to obtain BC with tunable mechanical properties, defined architectural patterns and additional functionalities, including responses to chemical, light or mechanical stimuli.

Post-2020 studies have further demonstrated that integrating genetic circuits with environmental sensing systems can lead to BC-based living materials capable of self-repair, self-regulation and chemical communication with their surroundings. In this context, BC is no longer viewed solely as a passive extracellular polymer, but as a programmable matrix that can host living cells and support dynamic functions over time.

In parallel, emerging approaches have explored the genetic modification of filamentous fungi used in mycelium-based materials. These strategies aim to control hyphal network density, extracellular polymer production, substrate colonisation and interactions with lignocellulosic residues. Although the genetic engineering of fungi for material applications still faces technical, regulatory and scalability challenges, recent studies suggest that manipulating fungal growth and differentiation pathways may lead to mycomaterials with more predictable mechanical, structural and functional properties (Caro-Astorga et al. 2021; Liu et al. 2025).

Together, these advances indicate that synthetic biology can transform BC- and mycelium-based materials from biologically derived matrices into programmable platforms. This transition is particularly relevant for the development of responsive, self-regenerating and multifunctional biocomposites.

### Concept and development of engineered living materials

Engineered living materials (ELMs) represent an emerging class of materials at the intersection of synthetic biology, materials engineering and biotechnology. They are characterised by the integration of structural material matrices with metabolically active living cells capable of self-organisation, environmental responsiveness and functional regeneration. Unlike conventional biomaterials, ELMs incorporate biological systems as active components of the material, thereby conferring dynamic properties that can evolve over time (Caro-Astorga et al. 2021; Liu et al. 2025).

Since 2020, several studies have explored ELMs based on genetically engineered single cells as well as microbial consortia. These systems allow the distribution of structural, metabolic and sensory functions within the same material platform. In BC-based ELMs, viable bacterial cells can be maintained within the cellulose matrix, enabling continuous material production, self-repair after physical damage and programmed responses to environmental changes such as pH variation, nutrient availability or the presence of contaminants (Lu et al. 2024; Pertile et al. 2025).

Hybrid ELMs involving fungal mycelium and other microorganisms have also attracted increasing attention. In these systems, the mycelial network provides structural support, mechanical integrity and substrate-binding capacity, while genetically modified bacteria or yeasts may perform active functions such as biosensing, localised biomolecule production, pollutant detection or environmental response. This division of labour makes microbial consortia particularly attractive for the design of robust, scalable and multifunctional living materials.

Despite their considerable potential, several challenges remain before ELMs can be broadly applied. These include maintaining long-term cellular viability, ensuring mechanical stability, controlling biological activity under variable environmental conditions and addressing biosafety and regulatory concerns associated with genetically modified organisms. In addition, the transition from laboratory-scale prototypes to reproducible and scalable material systems remains a key technological barrier.

Nevertheless, the convergence of synthetic biology, bacterial cellulose, fungal mycelium and engineered living materials is widely recognised as one of the most promising frontiers for the development of intelligent, sustainable and regenerative materials. By integrating structural performance with living functionality, these systems offer new possibilities for applications in biosensing, environmental remediation, smart packaging, biomedical devices and adaptive biocomposites.

### From Biofabricated matrices to programmable living systems

The progression from conventional biofabricated materials to ELMs reflects a broader conceptual shift in microbial biocomposite research. In traditional approaches, microorganisms are mainly used to produce extracellular matrices, such as BC or mycelial networks, which are subsequently harvested, processed and stabilised. In contrast, ELMs seek to preserve or engineer biological activity within the material itself, allowing the final structure to sense, respond, adapt or regenerate.

This transition is particularly relevant for BC and mycelium platforms because both already possess hierarchical organisation, high surface area, biocompatibility and compatibility with hybrid fabrication strategies. BC offers a hydrated nanofibrillar matrix suitable for hosting living cells, while fungal mycelium provides a naturally self-assembling, mechanically coherent and substrate-integrated network. When combined with synthetic biology, these properties enable the design of microbial materials with programmable architecture and active functionality.

Therefore, synthetic biology should not be viewed only as a tool for improving microbial production yields, but as a strategy to redefine the functional role of microorganisms in material systems. In this emerging framework, microbial cells act simultaneously as producers, organisers and functional components of the material. This perspective strengthens the relevance of BC- and mycelium-based biocomposites as platforms for the next generation of sustainable, intelligent and living materials.

### Advanced fabrication techniques

#### Additive manufacturing and 3D printing

Additive manufacturing, and particularly 3D printing, represents one of the most promising frontiers in the design and fabrication of functionalised biomaterials. Unlike traditional methods—such as moulding or simple extrusion —3D printing enables the construction of structures with highly complex geometries and millimetre-scale precision, built layer by layer and guided by computer-generated models. In this context, the incorporation of bio-inks containing bacterial cellulose (BC) and other biological matrices has proved fundamental for directing the morphological and mechanical properties of scaffolds, with direct potential applications in tissue engineering.

A recent example is the study that developed a 3D printing method for mycelium-based biocomposites, known as Mycofluid, which uses coffee husk residues and other organic substrates to print structures that can subsequently be colonised by fungal hyphae. This approach eliminates the need for rigid moulds and expands the capacity to create complex geometries directly using bioactive and compostable materials, demonstrating the feasibility of additive manufacturing of sustainable and structurally robust biomaterials (Luo et al. 2025).

Furthermore, more technically focused studies on bacterial cellulose (BC) indicate that, although pure BC presents high purity and biocompatibility for biomedical applications, its direct use in bio-inks faces processing challenges due to its highly entangled nanofibrillar network. Some approaches have incorporated BC as a reinforcing component within bio-printable hydrogels to overcome mechanical limitations and promote greater resistance to wear and deformation after printing, while maintaining the ability to form highly precise three-dimensional scaffolds (Choi et al. 2025).

The use of such BC-based bio-inks and other biocompatible polymeric matrices not only improves mechanical properties—such as elastic modulus and tensile strength—but also creates environments more favourable to cell adhesion and proliferation, which is crucial for precise tissue engineering and cellular regeneration (Choi et al. 2025).

### Multimaterial integration and hybrid systems

The integration of biological materials with synthetic or natural materials in multimaterial systems represents another advanced direction in additive manufacturing. Such strategies aim to combine complementary advantages from different classes of materials—for example, the intrinsic biocompatibility and sustainability of biological materials with the mechanical robustness and dimensional stability of conventional materials.

In the field of hybrid biocomposites involving mycelium, the study by Shen et al. (2024) presents a hybrid mycelium-based biocomposite platform in which the controlled colonisation of the fungal system within matrices produced by additive manufacturing substantially enhances mechanical properties, such as elastic modulus and tensile strength, while also enabling new functionalities including flexibility, conformability and water resistance. This research demonstrated the production of hybrid materials with a modulus of up to 160 MPa—an improvement of more than fifteenfold compared with conventionally moulded mycelium composites—and illustrated applications ranging from self-sealing containers to flexible biotextiles (Shen et al. 2024).

These hybrid systems not only expand the scope of applications — ranging from sustainable packaging to lightweight structural components — but also open pathways for the development of “living” structures capable of interacting with their environment. The combination of mycelium with conventional materials can be strategically explored to control internal properties such as hydrophobicity, compressive rigidity, and stimulus-responsive flexibility, enabling the creation of composites that redefine how we conceive bio-derived intelligent materials. Such systems highlight the potential of biologically derived composites to integrate adaptive functionality and structural performance, bridging the gap between biological growth processes and advanced materials engineering (Shen et al. 2024).

Furthermore, the integration of bacterial cellulose (BC) with other biopolymers and conventional materials can generate hybrid scaffolds with properties that can be tailored through composition and physical arrangement—for example, by improving porosity or structural interconnectivity, which is essential for nutrient diffusion in cell culture environments (Choi et al. 2025).

### Applications in strategic sectors

#### Biomedicine

Materials based on bacterial cellulose (BC) and its composites, including hybrids with mycelium or bioactive polymers, have been widely studied for a range of biomedical applications due to their unique properties—such as biocompatibility, highly porous structure, mechanical strength and ease of functional modification. These characteristics make BC a promising candidate for several applications:

- Scaffolds and tissue engineering: BC and bacterial nanocellulose (BNC) can serve as porous structures that support cell adhesion, proliferation and differentiation in bone and tissue regeneration applications (e.g., nanocellulose systems functionalised with mineral components such as hydroxyapatite), demonstrating potential for guided bone regeneration and matrices for cellular growth in tissue engineering (Gaulke et al. 2023).
-  Advanced wound dressings: Clinical and preclinical studies have investigated BC as a wound dressing material for chronic wounds, burns and lesions due to its capacity to maintain adequate moisture, as well as its biocompatibility and low toxicity (Esmail et al. 2025; Wahid et al. 2021).
- Controlled drug delivery and antibiofilm activity: Nanocellulose-based composites incorporating antimicrobial agents (e.g., chlorhexidine, amoxicillin, silver nanoparticles or zinc oxide) have demonstrated efficacy against bacterial biofilms and are being explored for drug delivery systems with targeted action in infection treatments or regenerative therapies (Kressin et al. 2026).
- Sequential delivery systems: Although not strictly based on BC, three-dimensional tissues and scaffolds fabricated from biopolymers have demonstrated the possibility of controlled and sequential release of therapeutic agents, a concept that may be integrated with bacterial nanocellulose for combined therapeutic strategies (Da Silva Pereira, 2024; Ahmad et al. 2021).
- Additional biomedical platforms: Reviews also highlight the use of BC in cell encapsulation, tumour culture models, support matrices for biopharmaceuticals and smart drug delivery devices, further expanding its potential for clinical applications and regenerative therapies (Liu et al. 2020; Wang et al. 2026).

### Sustainable packaging and products

BC and composite materials involving mycelium (fungal growth) are important candidates in the transition towards sustainable materials, particularly within the context of the circular economy and the replacement of petrochemical polymers:

- Biodegradable packaging: BC has been studied as an alternative to conventional plastics in food packaging and packaging for sensitive products due to its biodegradability, excellent mechanical strength and gas/moisture barrier properties. Recent studies indicate that BC-based films can reduce oxygen permeability and extend the shelf life of perishable products (Turganova et al. 2025).
- Active and intelligent technologies: BC compositions incorporating nanoparticles or natural additives (such as zinc oxide, silver or antioxidant compounds) can provide antimicrobial and sensing properties, paving the way for active packaging that improves food safety and monitors product quality (Deleanu et al. 2024).
- Fully biological materials based on mycelium: Mycelium-based composites, often combined with plant residues or BC, are being explored for compostable packaging and environmentally friendly consumer products (such as insulation materials, structural blocks and decorative components) that can replace synthetic foams and traditional polymeric materials (Majib et al. 2023, 2025).
- Sustainability and circular economy: The use of BC produced from fermentative cultures and mycelium grown on residual substrates (agro-industrial residues) exemplifies strategies for the valorisation of industrial waste and the integration of sustainable processes in materials manufacturing, reducing both production costs and environmental impact (Kniep et al. 2025).

Recent advances highlight the growing number of studies investigating bacterial cellulose and its composites for biomedical technologies and sustainable materials. Table 4 summarises representative studies published between 2020 and 2025, including materials used and the main functional outcomes.

**Table 4:** – Key articles (2020–2025) on biomedical and sustainable packaging applications based on bacterial cellulose (BC) and its composites, including the respective materials and main findings

Sector / Application	Type of Material	Main Result	Reference
Biomedicine – Antimicrobial wound dressings	BC nanofibrils with silver nanoparticles	Film exhibiting strong antibacterial activity for wound dressing applications	Zeng et al. 2023
Biomedicine – General wound healing	Functionalised BC	Review on functional modification strategies and applications in wound healing	He et al. 2021
Biomedicine – BC films with Ag/Chitosan	BC–CTS + Ag nanoparticles	Antibacterial film with good biocompatibility	Zhao et al. 2022
Packaging – BC–ZnO films for food	SA/BCNFs + ZnO nanoparticles	Biodegradable films with antioxidant and antibacterial activity	Zheng et al. 2025
Biomedicine – Reviews on wound dressings	BC nanocellulose for wound dressings	Trends and perspectives in BC-based wound dressing materials	Pasaribu et al. 2024
Biomedicine – BC in wound healing	BC micro- and nanofibres	Comprehensive review on BC for wound healing applications	Ahmed et al. 2020
Packaging – BC films for food packaging	BC film	Biodegradable film with good barrier properties for food packaging	Deng et al. 2024

Recent experimental studies and critical analyses of the literature indicate that materials based on bacterial cellulose (BC) and biological composites, including hybrid systems with mycelium and other biopolymers, are not limited to meeting environmental requirements such as biodegradability and compostability. They also achieve physical, mechanical and functional performance comparable to — and in some cases superior to — that of conventional petroleum-derived plastics. Studies published after 2020 demonstrate that BC films and structures exhibit high tensile strength, excellent dimensional stability under humid conditions, low gas permeability and good processability, attributes that are essential for industrial applications in packaging, lightweight construction and consumer products (Jiang et al. 2024; Teixeira et al. 2024).

In addition, the functionalisation of BC and mycelium-based composites has enabled the incorporation of advanced properties such as antimicrobial activity, antioxidant behaviour, responsiveness to environmental stimuli and thermal insulation, significantly expanding the range of possible applications compared with traditional synthetic polymers (Hamedi et al. 2020; Chen et al. 2025a, b, 2026).

In particular, hybrid systems incorporating mycelium have demonstrated favourable strength-to-weight ratios and tunable mechanical performance, positioning them as viable alternatives to synthetic foams and filler materials widely used in industry (Früchtl et al. 2023; Siche et al. 2023).

From a strategic perspective, these advances reinforce the role of BC and biological composites as high-impact industrial materials capable of integrating technical performance, environmental sustainability and alignment with circular economy principles. However, the literature also highlights that the full potential of these materials will only be realised when challenges related to scalability, standardisation of properties and economic feasibility are systematically addressed. This indicates that the functional superiority observed at laboratory scale must be accompanied by robust industrial production strategies and life-cycle assessments in order to consolidate their large-scale adoption.

### Industrialisation, regulation and technical challenges

Despite the significant advances in the development of materials based on bacterial cellulose (BC), mycelium and bioactive hybrid systems, the transition from laboratory research to industrial-scale production still faces substantial technical, regulatory and economic challenges. Among the main obstacles are the scalability of production processes, the biological variability inherent to living systems, and the absence of consolidated regulatory frameworks for living or partially living materials.

#### Scalability and process standardisation

The large-scale production of BC and mycelial composites is strongly influenced by factors such as cultivation conditions, culture medium composition, microbial strains and fermentation parameters, which can result in variations in the mechanical, structural and functional properties of the final material. Recent studies emphasise that industrial reproducibility requires the rigorous standardisation of fermentation protocols, including precise control of pH, oxygen supply, temperature and substrates, as well as the implementation of real-time monitoring strategies (Villarreal-Soto et al. 2021; Pertile et al. 2025).

In addition, emerging processes such as 3D printing, sheet-laminated manufacturing and guided biofabrication introduce new complexities related to bioink viscosity, dimensional stability and the integration between biological growth and additive manufacturing, requiring hybrid models of process engineering (Khan et al. 2022).

### Biological variability and quality control

Biological variability represents a critical challenge, particularly in mycelium-based materials or hybrid living systems. Different species, and even strains of the same fungus, may exhibit distinct growth rates, mycelial architecture and mechanical performance, making it difficult to obtain products with consistent specifications.

Recent studies on mycelium composites emphasise the need for rational strain selection, substrate engineering and post-growth treatments (drying, pressing and thermal inactivation) in order to ensure the stability and safety of the final product (Jones et al. 2020; Chen et al. 2025a, b; Hausner et al. 2025).

In the case of living or semi-living materials, controlling residual metabolic activity, cell viability and contamination risks becomes a central aspect for industrial and biomedical applications (Houette et al. 2022; Pertile et al. 2025).

#### Regulation and genetically modified materials

The regulation of biological and biofabricated materials remains fragmented and poorly harmonised internationally, particularly when genetically modified organisms (GMOs) or living materials are involved.

In biomedical applications, bacterial cellulose (BC)-based wound dressings and scaffolds already have regulatory precedents. However, hybrid composites or living materials challenge traditional classifications between biomaterials, medical devices and biotechnological products (Houette et al. 2022).

In the context of packaging and consumer products, issues related to biosafety, controlled biodegradation, metabolite release and environmental impact must be evaluated in an integrated manner. This reinforces the importance of life-cycle assessments (LCA) and risk analyses from the earliest stages of material development.

#### Life-cycle assessment and industrial feasibility

Life-cycle assessment (LCA) studies indicate that bacterial cellulose (BC)- and mycelium-based materials may offer environmental advantages over some petroleum-derived, mineral-based or conventional insulation materials. However, these advantages are highly dependent on the production scenario, functional unit, substrate, energy source, cultivation strategy, downstream processing and end-of-life assumptions. Therefore, BC- and mycelium-based materials should not be described as intrinsically sustainable; their environmental performance must be demonstrated under clearly defined process conditions (Forte et al. 2021; Alaux et al. 2024; Volk et al. 2024).

For BC, the main environmental hotspots are usually associated with culture medium composition, low fermentation productivity, energy demand during cultivation, water consumption, downstream washing/purification and chemical use. Forte et al. (2021) applied a cradle-to-gate LCA to BC production by static culture and showed that the production system still presents relevant environmental burdens, especially because of the resources required to obtain dry BC. More recent assessments comparing BC pulp and BC-derived filaments with plant-based cellulosic sources also reinforce that BC sustainability depends strongly on the production route and downstream processing requirements (Silva et al. 2025). These findings support the use of low-cost culture media, preferably derived from agro-industrial residues, as a strategy to reduce both production costs and environmental impacts. Nevertheless, the use of residues does not automatically guarantee better performance, because pretreatment, transport, variability in composition and purification requirements may also increase environmental burdens.

For mycelium-based composites, LCA studies also show promising but scenario-dependent results. Volk et al. (2024) reported that hemp-based lightweight mycelium composites produced in a laboratory-scale German model presented a climate-change impact of approximately 0.3668 kg CO₂-eq kg⁻¹ material, with advantages in climate-change impact and fossil-energy demand compared with several conventional insulation materials. However, the same study showed that electricity consumption during production and hemp cultivation were important contributors to environmental impact, and that land-use and water-demand indicators may be higher than those of some conventional alternatives (Volk et al. 2024). Similarly, Alaux et al. (2024), in a prospective cradle-to-grave LCA of mycelium-based insulation blocks, demonstrated that the environmental potential of these materials depends on assumptions related to cultivation, drying, transport, service life, biogenic carbon accounting and end-of-life treatment. These results indicate that mycelium materials may be environmentally favourable in specific scenarios, but their benefits are not universal.

From an industrial perspective, LCA evidence suggests that BC- and mycelium-based materials become more feasible when they are integrated into circular and resource-efficient bioprocesses. For BC, industrial adoption requires improvements in fermentation productivity, replacement of refined sugars and expensive culture media, reduction of water-intensive purification steps, and development of scalable bioreactor configurations (Forte et al. 2021; Silva et al. 2025). For mycelium-based composites, competitiveness depends on the use of locally available lignocellulosic residues, low-energy cultivation, efficient drying, controlled substrate quality, adequate mechanical and thermal performance, and realistic end-of-life strategies (Alaux et al. 2024; Volk et al. 2024). Consequently, future studies should combine LCA with techno-economic assessment, pilot-scale data and sensitivity analyses for electricity source, substrate transport, water reuse, drying technology and product durability. Only under these conditions can claims of environmental superiority over synthetic polymers, plant-derived cellulose materials or mineral insulation products be robustly supported.

#### Conclusions and future perspectives

Microbial biocomposites should not be viewed merely as substitutes for plastics or plant-derived composites. Their most disruptive feature is the use of microorganisms as active fabrication agents, capable of growing, self-organising and depositing structural biopolymers under mild conditions. In bacterial cellulose systems, for example, *Komagataeibacter* spp. secrete highly pure cellulose nanofibrils that form hydrated three-dimensional networks, and recent studies show that this biosynthesis can be spatially guided. Bacterial cellulose spheroids have been proposed as modular building blocks for 3D and patterned living materials, shifting the field from simply harvesting cellulose pellicles to programming geometry, assembly and functionality during material formation.

Synthetic biology further expands this concept by enabling microbial systems to produce materials that are not only structural, but also functional. Engineered bacteria or microbial co-cultures can be designed to incorporate sensing, pigmentation, enzymatic activity, chemical responsiveness or regenerative capacity directly into the material. This is particularly disruptive because production and functionalisation can occur in a single bioprocess, reducing the need for dyeing, coating or chemical post-treatment. In parallel, mycelium-based biocomposites exploit fungal hyphae as biologically grown reinforcing networks that colonise lignocellulosic residues and bind them into porous, cohesive structures, allowing low-temperature fabrication from agro-industrial waste.

The industrial novelty of microbial biofabrication lies in the convergence of biological self-assembly, digital design and process engineering. Additive manufacturing, templating, mould-based growth and bioprinting can guide bacterial cellulose deposition or fungal colonisation into predefined geometries, improving control over shape, porosity and architecture. However, translation to industry still requires solving bottlenecks related to productivity, growth reproducibility, contamination control, drying, dimensional stability, mechanical variability and biosafety, especially for living or genetically engineered materials. Therefore, the next disruptive step will be the development of programmable microbial systems, controlled consortia, scalable bioreactors or moulds, and predictive models linking growth conditions to final material properties. 

## Data Availability

No datasets were generated or analysed during the current study.

## Abbreviations

BC: Bacterial cellulose
BNC: Bacterial nanocellulose
MBCs: Mycelium—based composites
ELMs: Engineered living materials
GO: Graphene oxide
HAp: Hydroxyapatite
PLA: Polylactic acid
PHB: Polyhydroxybutyrate
PP: Polypropylene
LCA: Life—cycle assessment
TEMPO: 2,2,6,6—Tetramethylpiperidine—1—oxyl
c: di—GMP—Cyclic diguanylate monophosphate
UDP: glucose—Uridine diphosphate glucose
CRISPR/Cas9: Clustered regularly interspaced short palindromic repeats/CRISPR—associated protein 9
GMO: Genetically modified organism
3D: Three—dimensional
PVA: Poly(vinyl alcohol)
NaOH: Sodium hydroxide
ZnO: Zinc oxide
Ag: Silver
CO₂: eq—Carbon dioxide equivalent
kg CO₂: eq—Kilograms of carbon dioxide equivalent
µm: Micrometre
MPa: Megapascal
kGy: Kilogray
w/v: Weight per volume
pH: Potential of hydrogen
